# Effects of Combined Blood Flow Restriction and Inter‐Effort Recovery Hypoxia On Repeated‐Sprint Performance And Physiological Responses: A Randomized And Single‐Blinded Crossover Trial

**DOI:** 10.1002/ejsc.70284

**Published:** 2026-11-05

**Authors:** Carlos Dellavechia de Carvalho, Germano Marcolino Putti, Mohammed Ihsan, Brendan Richard Scott, Peter Peeling, Marcelo Papoti, Olivier Girard

**Affiliations:** ^1^ Ribeirao Preto Medical School University of Sao Paulo Ribeirao Preto Brazil; ^2^ School of Human Sciences (Exercise and Sport Science) The University of Western Australia Perth Australia; ^3^ Hypoxia, Sport and Health Team, School of Applied Sciences State University of Campinas Limeira Brazil; ^4^ School of Physical Education and Sports of Ribeirao Preto University of Sao Paulo Ribeirao Preto Brazil; ^5^ Physical Education Department College of Education United Arab Emirates University Al Ain UAE; ^6^ Physical Activity, Sport and Exercise (PHASE) Research Group, School of Allied Health (Exercise Science) Murdoch University Perth Australia; ^7^ Centre for Healthy Ageing Murdoch University Perth Australia

**Keywords:** altitude, cardiorespiratory fitness, exercise test, ischemic preconditioning, near‐infrared spectroscopy

## Abstract

We investigated the acute effects of local and/or systemic hypoxia on performance and psycho‐physiological responses during repeated cycle sprints. Eleven physically active young adults (six men, five women) performed four sessions on separate days, each involving three sets of five 5‐s all‐out efforts on a cycle ergometer (25 seconds rest between efforts and 5 minutes rest between sets). Sessions were conducted under four conditions, combining blood flow restriction [BFR; 45% of arterial occlusion pressure (AOP)] or control (CON; 5% of AOP) applied bilaterally to the legs during sprints, with inter‐effort recovery hypoxia [IEH; inspired oxygen fraction (FiO_2_) = 13.5%] or normoxia (NOR; FiO_2_ = 20.9%). Power output decreased across sprints and heart rate increased over sets, with no differences between conditions (Δ = −9.18%). IEH resulted in lower peripheral oxygen saturation (Δ = −8.0%) but higher oxygen uptake (Δ = 6.7 mL·kg^−1^ min^−1^) and minute ventilation (Δ = 6.1 L·kg^−1^ min^−1^), compared to normoxic conditions. Blood lactate concentration increased (Δ = 6.5 mM) post‐session similarly across all conditions. Vastus lateralis muscle oxygenation during sprints and inter‐set recovery was comparable across conditions, although the mean tissue saturation index during inter‐set recovery was lower in BFR + IEH versus CON + NOR conditions (Δ = −4.0%). Ratings of perceived exertion (Δ = 22.5%), limb discomfort (Δ = 34.2%), and breathing difficulty (Δ = 43.7%) increased throughout the sets, independently of conditions. Despite physiological and perceptual adjustments across sprint repetitions, neither BFR during sprints nor systemic hypoxia during recovery altered mechanical output in physically active individuals.

## Highlights

- Inter‐effort recovery hypoxia increases ventilatory responses and decreases peripheral oxygen saturation and mean tissue saturation index during recovery in repeated‐sprint exercise.
- Blood flow restriction during efforts in a repeated‐sprint exercise session negatively affects peak power output, and when combined with inter‐effort recovery hypoxia, it reduces the tissue saturation index.
- The combination of local hypoxia during efforts and systemic hypoxia during recovery does not result in a cumulative reduction in performance in repeated‐sprint exercise.

## 1 Introduction

Repeated‐sprint ability (RSA) refers to the capacity to produce the best possible average performance over multiple short (≤ 10 seconds) and ‘all‐out’ efforts separated by brief (≤ 60 seconds) recovery periods (Girard et al. 2011). This fitness component is essential for most team‐sport athletes due to the high‐intensity, intermittent nature of competition (Bishop et al. 2011; Girard et al. 2011). Performance during repeated‐sprint exercise (RSE) typically declines due to reduced muscle excitability, impaired energy supply, metabolite accumulation, and/or decreased neural drive to active musculature (Girard et al. 2011). Training interventions that target these limiting factors can boost RSA (Bishop et al. 2011).

To maximize adaptations from high‐intensity training, RSE has been combined with systemic hypoxic exposure (Girard et al. 2017). Faiss et al. (2013) showed larger improvements in repeated‐sprint performance after only eight RSE sessions in normobaric hypoxia compared with normoxia. However, recent findings suggest that continuous systemic hypoxia during training can impair RSA (Li et al. 2024; Raberin et al. 2023). As an alternative, intermittent systemic hypoxia, applied during specific (active or passive) phases of RSE, has been proposed to maintain training quality while still providing a hypoxic stimulus. Inter‐effort recovery hypoxia (IEH) (Papoti et al. 2023), where systemic hypoxia is applied only during recovery intervals, has been shown to preserve performance in high‐intensity interval training sessions (Dellavechia de Carvalho, Putti, et al. 2023; Putti et al. 2026; Foresti et al. 2023). In support of this finding, Li et al. (2024) showed that IEH maintains power output comparable to normoxia while eliciting lower peripheral oxygen saturation and outperforming continuous hypoxia in both peak and mean power output.

In addition to systemic hypoxia, local hypoxia induced by blood flow restriction (BFR) has recently been investigated in RSE protocols (Giovanna et al. 2022; Ienaga et al. 2022; Kojima et al. 2021). BFR may amplify psychophysiological responses to exercise through greater metabolite accumulation, impaired oxygen delivery, and enhanced activation of group III/IV muscle afferents, which have been associated with increased perceptions of effort and muscle discomfort (Bielitzki et al. 2024). Similar to systemic hypoxia, continuous BFR application during RSE has been shown to impair performance (Willis et al. 2018; Willis et al. 2019). As an alternative, intermittent RSE application has been proposed. Both Ienaga et al. (2022) and Kojima et al. (2021) showed that applying BFR only during recovery period augments muscle deoxygenation without affecting mechanical output. More recently, Mckee et al. (2023) investigated local hypoxia applied during discrete RSE phases and showed that BFR only during sprints did not impair performance, while continuous or recovery‐only BFR compromised power output.

Both systemic and local hypoxia (i.e., BFR) reduce oxygen availability in exercising muscles (Willis et al. 2019), and their combination may amplify physiological responses. Girard et al. (2019) first investigated this in resistance exercise (70% of 1RM to concentric failure) and found no effect of local, systemic, or combined hypoxic stimuli on the number of repetitions. Early studies applying both stimuli during RSE revealed that continuous BFR or systemic hypoxia impaired performance, with their combined use further exaggerating this decline (Willis et al. 2019; Willis, Borrani, and Millet 2019). These findings suggest concurrent application of both stressors during the same RSE phases may be counterproductive. However, Wang et al. 2023 showed that applying BFR during recovery under systemic hypoxia increased total hemoglobin (i.e., indicating increased blood pooling) and augmented muscle deoxygenation without compromising performance, although BFR was not applied throughout the entire recovery period.

Different approaches exist for combining local and systemic hypoxia, though some impair performance. Mckee et al. (2023) showed that applying BFR only during efforts did not hamper performance, similar to the IEH method, where systemic hypoxia is applied exclusively during recovery periods (Botta de Arruda et al. 2025; Li et al. 2024). This suggests that the combination of BFR during efforts and systemic hypoxia during recovery intervals could be a promising strategy to enhance physiological stress without compromising mechanical output. This additional physiological stress, achieved without compromising sprint mechanical output, may enhance the adaptive stimulus during training and contribute to chronic improvements in aerobic capacity and oxygen utilization with both BFR (Taylor et al. 2016) and IEH (Putti et al. 2026; Roels et al. 2005) compared with normoxic training.

This study assessed the separate and combined acute effects of BFR during sprints and IEH on performance and psycho‐physiological responses during RSE. We hypothesized that BFR would accentuate local muscle deoxygenation and exercise‐related sensations, whereas IEH would reduce peripheral oxygen saturation (SpO_2_) and alter oxygen uptake during between‐set recoveries. We also expected that combining BFR and IEH would amplify psycho‐physiological responses without compromising mechanical performance.

## 2 Methods

### 2.1 Participants

An a priori power analysis using G*Power (version 3.1, Düsseldorf, Germany) determined that 11 participants were required to achieve 99% power at *α* = 0.05, based on an effect size of 0.59 from Willis et al. (2019), who reported significant group‐by‐time interactions on sprint number and mean power when comparing BFR and systemic hypoxia combinations with controls. Inclusion criteria were: age = 18–40 years, being physically active, and having not traveled above 1500 m in the six months before the experiment. Exclusion criteria were: getting injured or sick during intervention and not completing one of the four experimental sessions. Eleven (6 men and 5 women) physically active young adults (age = 30.9 ± 4.1 years, height = 170.0 ± 6.3 cm, body mass = 72.0 ± 10.8 kg, training frequency = 5 ± 2 sessions; training volume = 399.5 ± 284.9 min/week in their current typical week) volunteered for the study. All participants completed the Exercise & Sports Science Australia (ESSA) pre‐exercise screening and provided written consent. The study was approved by The University of Western Australia Human Research Ethics Committee (2023/ET000791).

### 2.2 Experimental Design

All sessions were conducted at UWA Exercise & Performance Centre, located at sea level. This study employed a single‐blinded, within‐participant, randomized crossover design. Participants completed one familiarization session followed by four randomized (using an electronic random number generator) session, at least 2 days apart. Each session involved performing an RSE under one of four conditions combining two levels of BFR [5% (sham protocol) and 45% AOP during sprints] with two inter‐effort hypoxia levels (FiO_2_ = 20.9% and 13.5%): 5% BFR with normoxia (CON + NOR), 5% BFR with hypoxia (CON + IEH), 45% BFR with normoxia (BFR + NOR), 45% BFR with hypoxia (BFR + IEH). Participants were instructed to avoid strenuous exercise, caffeine, and alcohol for 24 hours before testing and to arrive well‐rested and hydrated. During each session, participants wore a facemask, a pulse oximeter on one finger, and a wireless NIRS probe on the right vastus lateralis muscle. Primary outcomes included mean and peak power output and total work. Secondary measures were oxygen uptake, heart rate, SpO_2_, muscle oxygenation, and exercise‐related sensations.

### 2.3 Familiarization Session

Upon arrival, participants provided informed consent and completed Part 1 of the ESSA pre‐exercise screening. Anthropometric measurements (height, body weight, and upper thigh circumference) were collected using a stadiometer and electronic scale. Afterward, AOP was assessed with participants in the supine position using a 10‐cm pneumatic cuff (SC10, Hokanson, Bellevue, WA) placed proximally on the right thigh and a Doppler ultrasound probe (MD6 Doppler, Hokanson) positioned over the posterior tibial artery. The cuff was inflated using a rapid cuff inflator and AG101 air source (Hokanson E20, Bellevue, WA), and AOP was defined as the minimum pressure at which the arterial pulse was no longer detectable (Loenneke et al. 2011; Mckee et al. 2023). The warm‐up protocol, adapted from Mckee et al. (2023), included 5 minutes of cycling at 1kg^−1^, followed by four 5‐s sprints of 60%, 70%, 80%, and 90% of perceived maximum effort with 40 s of passive rest between each, ending with one ‘all out’ 5‐s sprint.

### 2.4 Experimental Design

Training sessions were adapted from Mckee et al. (2023). After the warm‐up (see above), participants rested passively for 5 minutes before starting the first set. Each session consisted of three sets of five 5‐s sprints, with 25 seconds of passive rest between sprints and 3 minutes between sets. Sprints were performed on a stationary cycle ergometer with an air‐brake resistance system. IEH was applied in four 5‐min blocks (before and after each set of sprints), while BFR was applied only during the 5‐s efforts (Figure 1).

**FIGURE 1:**
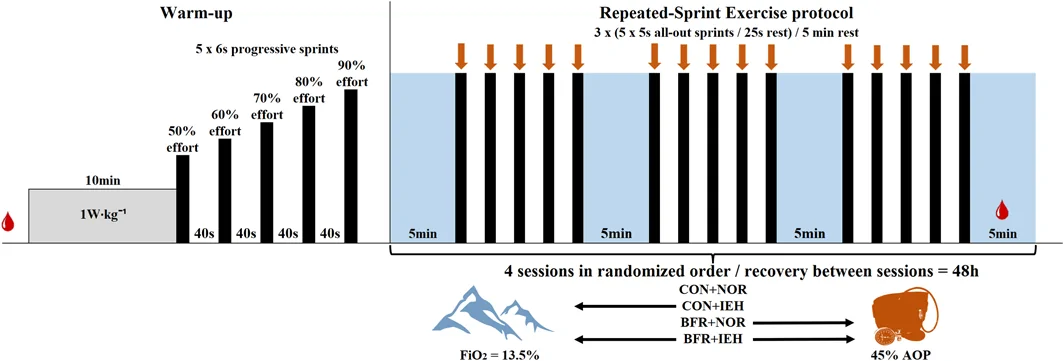
Overview of experimental sessions. AOP = arterial occlusion pressure; BFR = blood flow restriction; CON = control; FiO_2_ = fraction of inspired oxygen; IEH = inter‐effort recovery hypoxia; NOR = normoxia; blood droplets represent the blood collection; orange arrows represent the BFR cuff inflation.

### 2.5 Blood Flow Restriction

Occlusion was applied bilaterally to the lower limbs using an automated cuff inflation system (Hokanson E20, Bellevue, WA) with 10‐cm‐wide inflatable cuffs. During BFR conditions, cuff pressure was set to 45% of AOP (77.8 ± 9.8 mmHg), while the control condition used 5% of AOP (8.6 ± 1.1 mmHg). We chose this pressure (i.e., 45% of AOP) based on the study of Mckee, Girard, Peiffer, Hiscock, et al. (2024), that showed equivalent training adaptations between BFR and non‐BFR RST, but with ∼15% less power output (i.e., less work required) in the BFR group. Participants were blinded to the cuff pressure. Cuffs were inflated 1s before each sprint and deflated immediately after (Mckee et al. 2023).

### 2.6 Inter‐Effort Recovery Hypoxia

Participants wore a one‐way mask connected to a hose and reservoir bag, supplied by a portable hypoxia generator (F10 unit, The Altitude Box, Australia) that maintained inspired oxygen fraction (FiO_2_) between 13.0% and 13.5%. The mask was worn for 5 minutes before the first set of sprints, during recovery between sets, and for 5 minutes post‐exercise. For blinding purposes, control conditions followed the same procedure, but ambient air (FiO_2_ = 20.9%) was delivered via a valve system. FiO_2_ was continuously monitored using a Handi + oxygen monitor (Maxtec, Salt Lake City, Utah, US).

### 2.7 Mechanical Responses

All cycling tests were conducted on a custom‐made front‐access cycle ergometer (School of Sport Science, Exercise and Health; University of Western Australia), calibrated using the NATA Ergometer Calibration Rig (Biomed Electronic Services, Australia). Seat height, handlebar position, and gear range were individually adjusted during familiarisation and replicated in all trials. Mean power output (MPO), peak power output (PPO), and total work were recorded for each sprint at 1 Hz using customized software (Cyclemax, The University of Western Australia, Australia).

### 2.8 Physiological Responses

Oxygen uptake (VO_2_), minute ventilation, and respiratory rate were measured breath‐by‐breath using a Parvo TrueOne 2400 metabolic cart (Parvo Medics, Salt Lake City, USA). The system was calibrated before each test following manufacturer recommendations, using a 3 L syringe (5530 series; Hans Rudolph, Kansas City, USA) for flow calibration and a gas mixture (16.0% oxygen, 4.1% carbon dioxide) for gas analyzer calibration. Mean VO_2_ and ventilation were calculated during the 5 minutes preceding the first set and during recovery intervals between sets.

Heart rate and SpO_2_ were continuously monitored at 1 Hz via fingertip pulse oximetry (IVAC Vital Care DOX Model 506 DXNT, Criticare System Inc. USA). Mean values were calculated for each sprint set and the corresponding recovery periods.

Muscle oxygenation of the right vastus lateralis was continuously monitored using a wireless near‐infrared spectroscopy (NIRS) system (Portalite, Artinis Medical Systems, The Netherlands). The device features three emitter diodes at 30, 35, and 40 mm from the detector, emitting infrared light at 760 and 850 nm. Data from the 35 mm emitter‐detector distance (i.e., penetration depth ∼17.5 mm) were used for analysis. The NIRS probe was positioned on the vastus lateralis muscle approximately 12–15 cm proximal to the patella border. The probe location was marked and photographed to ensure consistent repositioning between conditions. The Portalite employs modified Beer–Lambert and spatially resolved spectroscopy to measure changes in oxygenated hemoglobin and deoxygenated hemoglobin (μM), as well as the tissue saturation index [TSI (%)] reflecting the dynamic balance between oxygen demand and supply in muscle microcirculation (Ferrari et al. 2004). Data were collected at 10 Hz using dedicated software (Oxysoft, Artinis Medical Systems) and down‐sampled to 1 Hz for further analyses. Within‐set muscle oxygenation profiles were assessed by TSI‐derived deoxygenation and reoxygenation amplitudes (Ihsan et al. 2013). Briefly, deoxygenation amplitude was the difference between the highest and lowest TSI at the start and end of deoxygenation, respectively. Reoxygenation amplitude was the difference between the lowest and highest TSI values at the end of deoxygenation and the start of sprint effort. Inter‐set recovery was assessed by mean TSI changes during recovery periods.

Capillary blood lactate concentration ([La]) was measured from earlobe samples using an automated analyzer (Lactate Pro LT‐1710, Arkray, Japan) before warm‐up and 3 minutes after the final sprint.

### 2.9 Perceptual Responses

Ratings of perceived exertion (RPE), sense of effort, perceived lower‐limb heaviness, and breathing difficulty were recorded using modified Borg CR10 scales (Christian et al. 2014). All measures were recorded immediately after each set of sprints in an invariant order.

### 2.10 Statistical Analysis

Data were analyzed using generalized linear mixed models. Outliers were initially screened using scatterplots and boxplots. Models included experimental condition, repeated‐measures factor (set, sprint, or recovery, depending on the variable), and their interaction as fixed effects, with participant included as a random intercept and sex as a covariate. Mean session HR and SpO_2_ were analyzed without a repeated‐measures factor, as only one value was obtained per session.

Missing data were handled using all available observations without imputation, such that only missing observations were excluded while all remaining participant data were retained. Because of the relatively small sample size and risk of model overfitting, peak power, mean power, and work were analyzed using two complementary approaches: (i) sprint‐level models within sets and (ii) set‐level models based on mean values. Although the data had a hierarchical structure (sprints nested within sets and participants), a more complex nested random‐effects structure was not implemented to preserve model stability and parsimony.

Model assumptions were evaluated through inspection of residual homoscedasticity (Pearson residuals vs. fitted values) and normality (histograms and Q–Q plots). Models were fitted using an identity link function assuming either Gamma or Gaussian distributions, and the best‐fitting model was selected using Akaike's Information Criterion (AIC). The selected distribution and final sample for each model are reported in the relevant figure or table legends.

Estimated marginal means and 95% confidence intervals were derived from the final models. Pairwise comparisons were adjusted with Bonferroni correction, with statistical significance set at *p* < 0.05. Effect sizes (d) were also calculated to facilitate comparison with previous literature and were interpreted as trivial (< 0.2), small (0.2–0.5), moderate (0.5–0.8), or large (> 0.8). Analyses were carried out in R (v 4.4.1) (R Core Team 2025), using the lme4 (v. 1.1.35.5) (Bates et al. 2015), ggResidpanel (v. 0.3.0) (Goode and Rey 2019), and emmeans (v. 1.10.3) (Lenth et al. 2025) packages.

## 3 Results

### 3.1 Performance Variables

#### 3.1.1 Experimental Design

Significant main effects of time were observed for PPO, MPO, and total work, and a significant main effect of condition was observed for PPO. Post hoc analysis revealed that the mechanical output declined across sprints within each set (*p* < 0.049; *d* > 0.871; Figure 2A–C). The peak power at Set 3 was lower in BFR + NOR and BFR + IEH compared to CON + NOR (*p* < 0.040; *d* > 0.802; Figure 2D).

**FIGURE 2:**
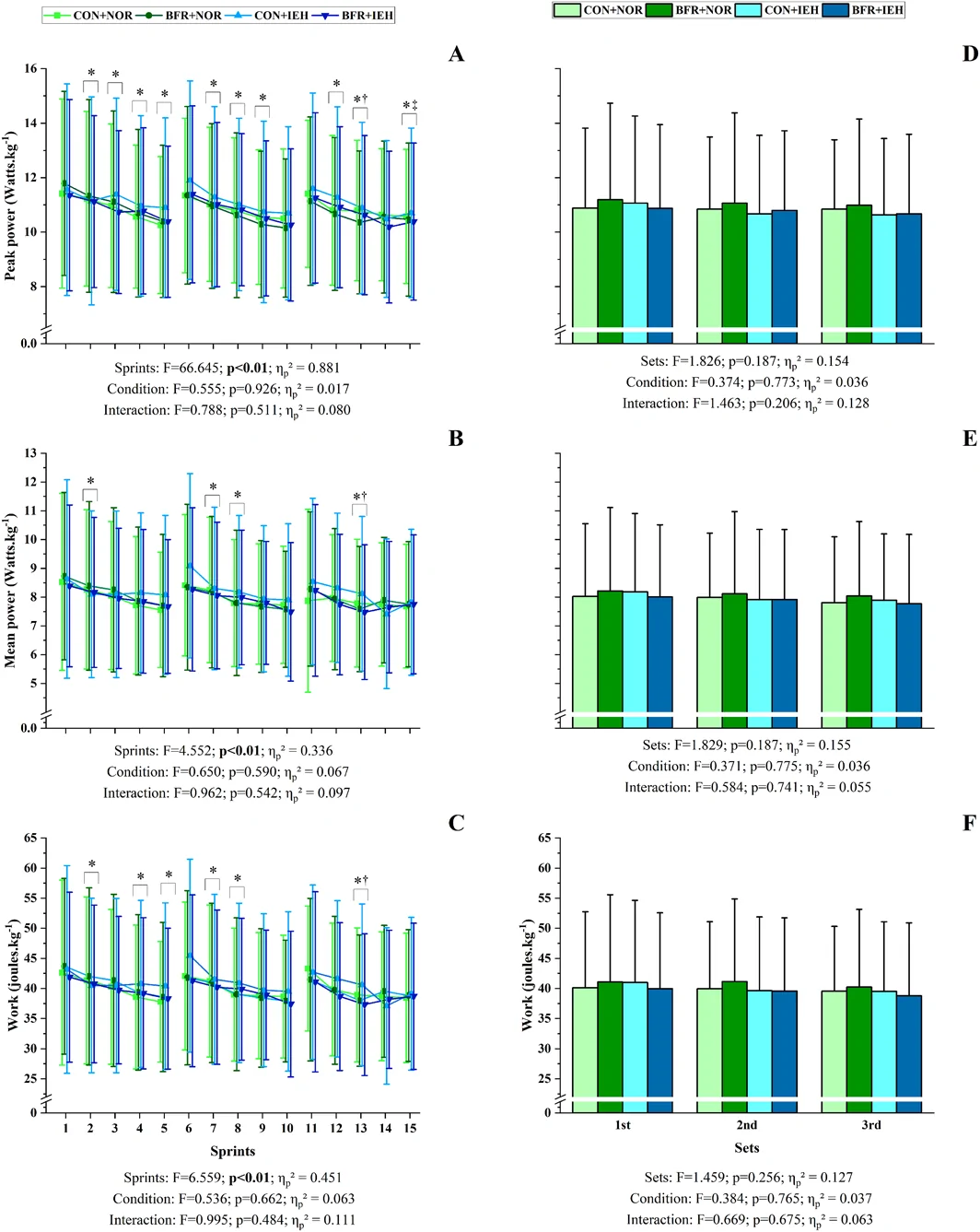
Peak power (A and D), mean power (B and E), and work (C and F) from each sprint (A–C), and each session during sets 1, 2, and 3 (D–F) of the repeated‐sprint exercise session. ∗ = significantly different from sprint 1; † = significantly different from CON + NOR; BFR = blood flow restriction; CON = control; IEH = inter‐effort recovery hypoxia; NOR = normoxia; ɵ = significantly different from sprint 2. Data are presented as estimated means and 95% confidence intervals from the generalized linear mixed model. The gamma distribution was used for peak and mean power in sprint comparison, and the Gaussian distribution was used for all other variables in the figure. *N* = 10 for peak power, mean power, and work in set 2, sprint 1, condition CON + IEH; for mean power and work in set 3, sprint 1, condition CON + NOR; *N* = 11 for all other variables.

### 3.2 Physiological Responses

For VO_2_, significant main effects were observed for set, condition, and their interaction. Post hoc analyses showed VO_2_ increased significantly after all sets compared to Baseline (*p* < 0.001; *d* > 1.559; Table 1). VO_2_ was consistently higher in IEH than NOR conditions (*p* < 0.001; *d* > 1.550; Table 1), except at Baseline (*p* = 0.058; *d* < 0.938; Table 1).

**TABLE 1:** Acute physiological responses during sets 1, 2, and 3 of repeated‐sprint exercise in the four conditions.

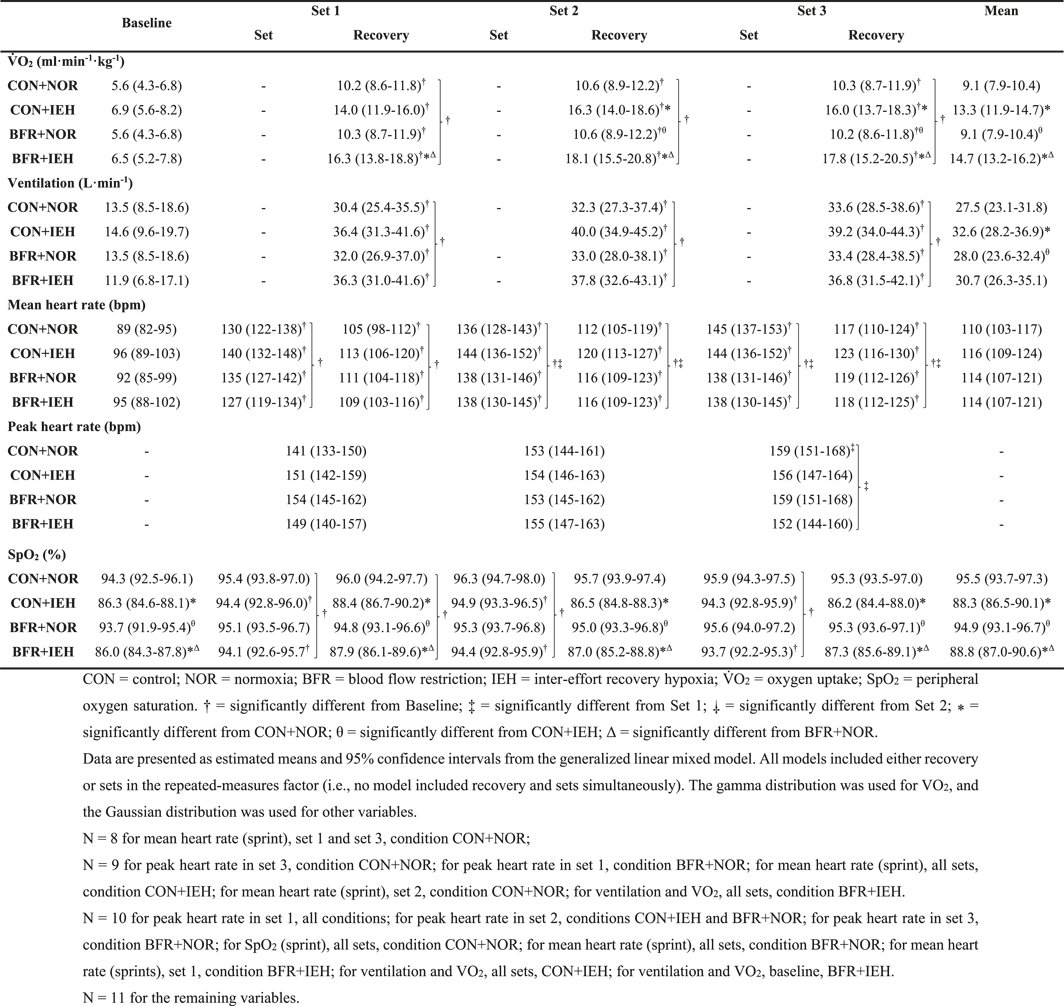

Minute ventilation showed significant main effects for set, condition, and interaction. Ventilation increased significantly after all sets compared to Baseline (*p* < 0.001; *d* > 2.632; Table 1). Additionally, ventilation was higher for CON + IEH compared to CON + NOR and BFR + NOR (*p* < 0.001; *d* > 1.213; Table 1).

Mean HR during sessions and peak HR showed significant main effects for set only. Mean HR was significantly higher during sets and during recovery compared to baseline (*p* < 0.002; *d* > 1.329; Table 1 and Figure 3), and higher in Sets 2 and 3 during sets and recovery compared to Set 1 (*p* < 0.031; *d* > 0.896; Table 1 and Figure 3). Peak HR was higher in Set 3 versus Set 1 for CON + NOR (*p* = 0.004; *d* = 1.387; Table 1 and Figure 3) and for all groups combined (*p* = 0.001; *d* = 1.146; Table 1 and Figure 3).

**FIGURE 3:**
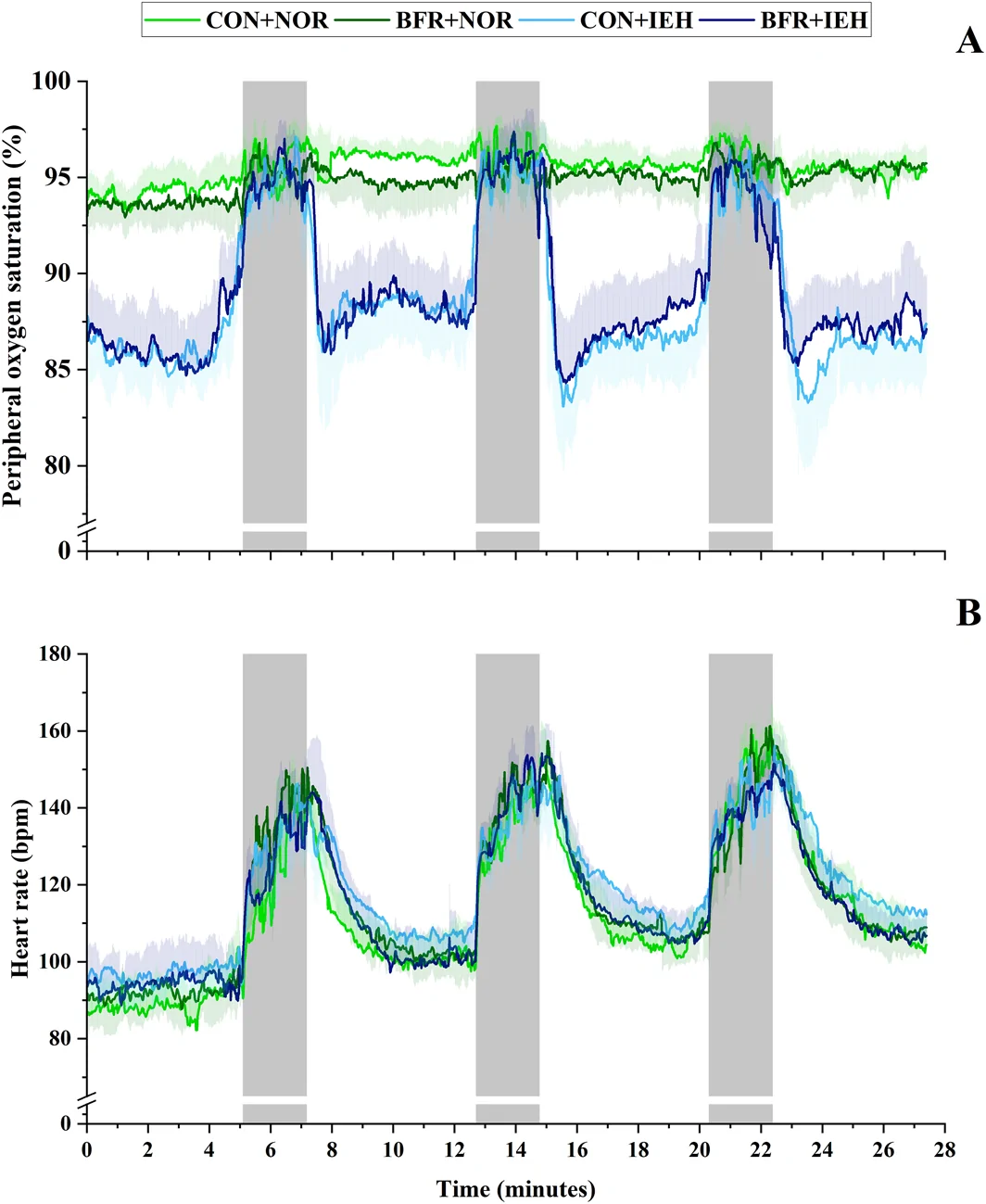
Peripheral oxygen saturation (A) and heart rate (B) during repeated‐sprint exercise sessions in the four conditions. BFR = blood flow restriction; CON = control; IEH = inter‐effort recovery hypoxia; NOR = normoxia. Gray areas represent the sprint sets. Data are presented as mean and 95% confidence intervals (*n* = 11).

For SpO_2_, significant main effects were observed for set, group, and interaction. Post hoc analyses indicated significantly lower SpO_2_ at baseline (*p* < 0.001; *d* > 2.611) and recovery (*p* < 0.001; *d* > 2.089) in IEH conditions compared to sets (Table 1 and Figure 3), and lower SpO_2_ in IEH versus NOR conditions (*p* < 0.001; *d* > 1.641; Table 1 and Figure 3).

A significant main effect of time was observed for [La], with post‐session values significantly higher than pre‐session across all conditions (*p* < 0.001; *d* = 2.272; Figure 4).

**FIGURE 4:**
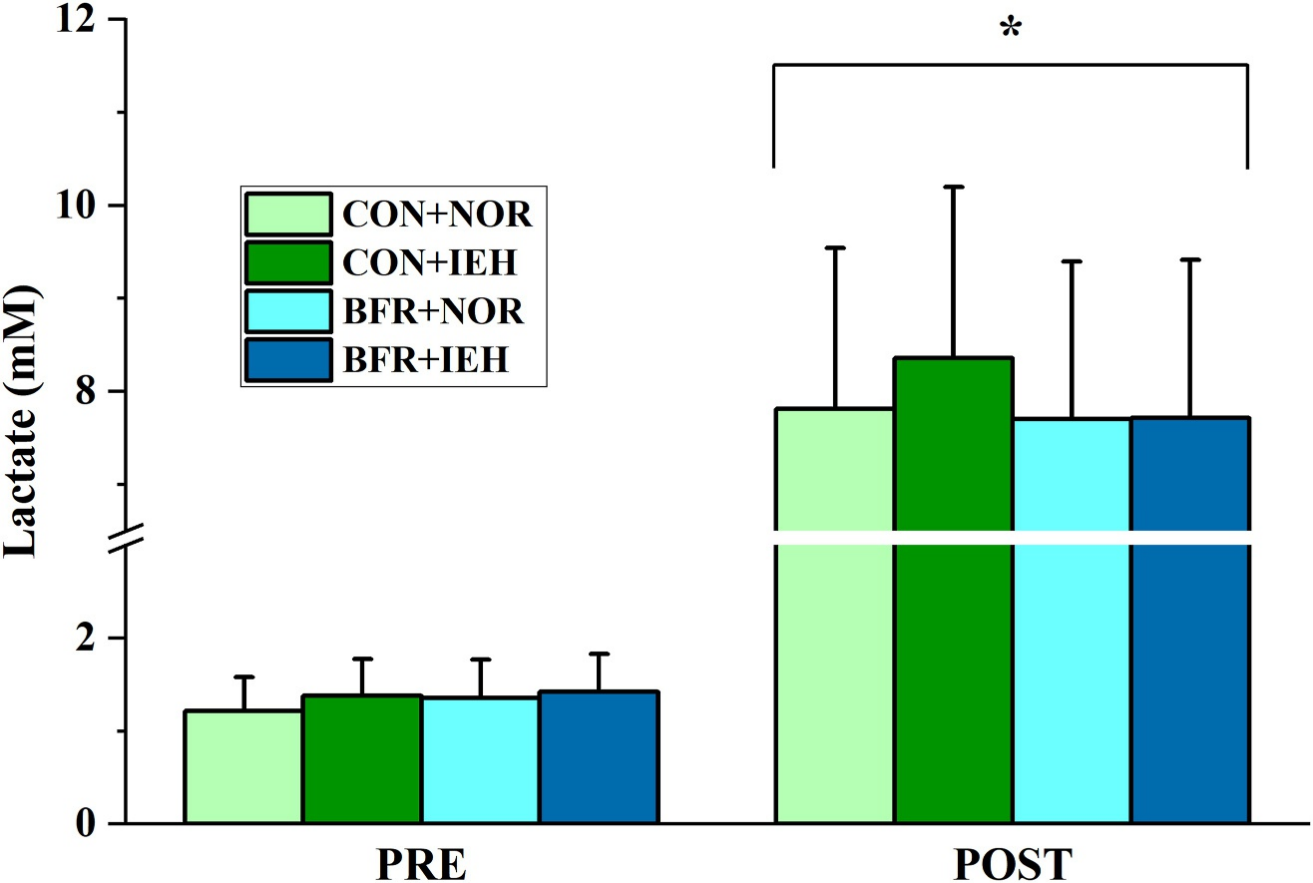
Blood lactate concentrations pre‐ and post‐repeated‐sprint exercise sessions under the four conditions. BFR = blood flow restriction; CON = control; IEH = inter‐effort recovery hypoxia; NOR = normoxia. Data are presented as estimated means and 95% confidence intervals from the generalized linear mixed model, with the Gamma distribution (*n* = 11).

### 3.3 Muscle Oxygenation

No significant effects were found for deoxygenation amplitude during sets (*F* < 1.456; *p* > 0.258; *η*_*p*_^2^ < 0.139) or reoxygenation amplitude during recovery periods (*F* < 2.923; *p* > 0.102; *η*_*p*_^2^ < 0.245) (Table 2 and Figure 5). A significant main effect for condition was observed for mean TSI during recovery. Post hoc analyses indicated significantly lower values for BFR + IEH compared to CON + NOR at Set 2 (*p* = 0.006; *d* = 1.179) and CON + NOR and BFR + NOR at Set 3 (*p* < 0.010; *d* > 1.139), while IEH conditions presented lower values (*p* < 0.001; *d* > 1.368) compared to NOR conditions (Table 2 and Figure 5).

**TABLE 2:** Tissue saturation index during sets 1, 2, and 3 of repeated‐sprint exercise in the four conditions.

	Set 1	Set 2	Set 3	Mean
Deoxygenation amplitude (%)				
CON + NOR	10.8 (7.2–14.5)	10.7 (7.0–14.3)	10.9 (7.3–14.6)	10.8 (7.2–14.4)
CON + IEH	10.9 (7.2–14.5)	11.3 (7.6–14.9)	10.6 (7.0–14.3)	10.9 (7.3–14.5)
BFR + NOR	11.3 (7.6–15.0)	10.8 (7.2–14.5)	10.8 (7.2–14.5)	11.0 (7.4–14.6)
BFR + IEH	10.7 (7.1–14.4)	10.8 (7.1–14.4)	11.4 (7.7–15.0)	11.0 (7.3–14.6)
Reoxygenation amplitude (%)				
CON + NOR	10.1 (6.8–13.5)	9.8 (6.5–13.2)	9.4 (6.1–12.7)	9.8 (6.5–13.1)
CON + IEH	10.2 (6.8–13.5)	10.1 (6.8–13.4)	9.6 (6.3–12.9)	10.0 (6.7–13.2)
BFR + NOR	10.4 (7.1–13.8)	10.2 (6.9–13.6)	9.8 (6.5–13.1)	10.2 (6.9–13.4)
BFR + IEH	10.0 (6.6–13.3)	10.0 (6.7–13.3)	9.9 (6.5–13.2)	9.9 (6.7–13.2)
Mean TSI during recovery (%)				
CON + NOR	75.4 (71.3–79.4)	76.7 (72.7–80.8)	75.4 (71.3–79.4)	75.8 (72.0–79.7)
CON + IEH	72.9 (68.9–77.0)	73.4 (69.3–77.4)	72.6 (68.6–76.6)	73.0 (69.1–76.8)^a^
BFR + NOR	75.7 (71.6–79.8)	76.1 (72.0–80.2)	75.9 (71.8–80.0)	75.9 (72.0–79.8)^b^
BFR + IEH	72.2 (68.1–76.2)	72.5 (68.4–76.5)^a^	71.3 (67.3–75.4)^a^ ^,^ ^c^	72.0 (68.1–75.8)^a^ ^,^ ^c^

*Note:* Data are presented as estimated means and 95% confidence intervals from the generalized linear mixed model. The gamma distribution was used for all variables. *N* = 10 for deoxygenation and reoxygenation amplitude, set 1, condition CON + NOR. *N* = 11 for all other variables.

Abbreviations: BFR = blood flow restriction; CON = control; IEH = inter‐effort recovery hypoxia; NOR = normoxia.

^a^ significantly different from CON + NOR.

^b^ significantly different from CON + IEH.

^c^ significantly different from BFR + NOR.

**FIGURE 5:**
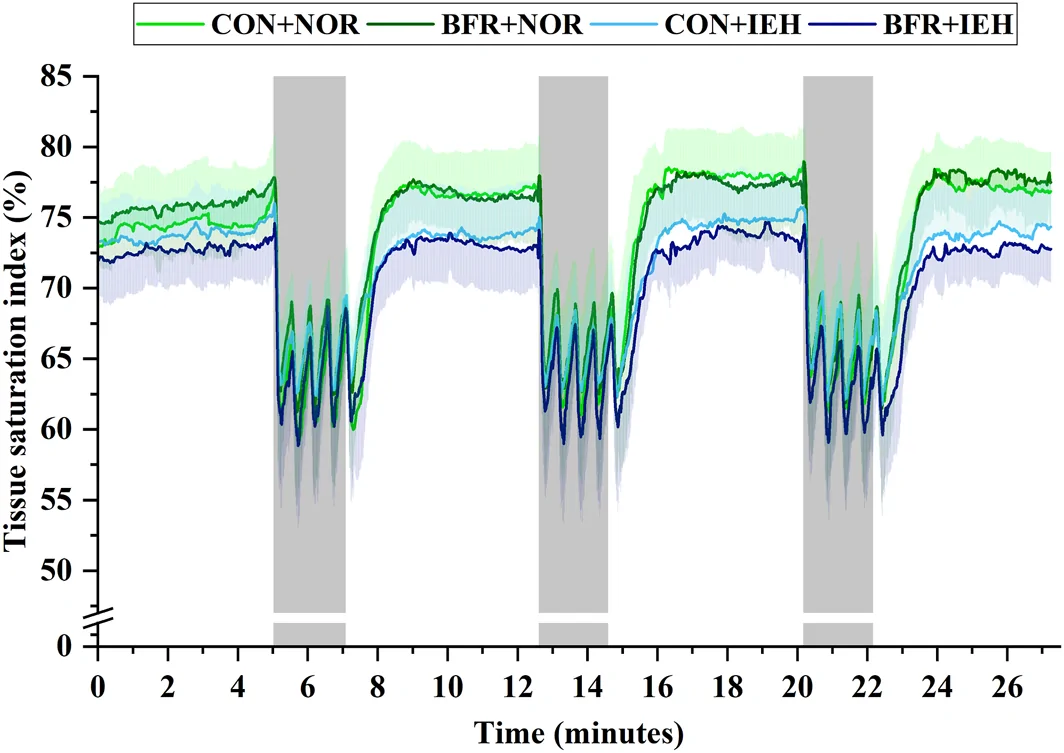
Tissue saturation index during repeated‐sprint exercise sessions in the four conditions. BFR = blood flow restriction; CON = control; IEH = inter‐effort recovery hypoxia; NOR = normoxia. Data are presented as mean and 95% confidence intervals (*n* = 11).

### 3.4 Perceptual Responses

Significant main effects for set and condition were found for RPE. Post hoc analyses indicated that RPE increased as the sets progressed (*p* < 0.042; *d* > 0.750; Table 3), and it was higher in BFR + NOR than in CON + NOR (*p* = 0.049; *d* = 0.811; Table 3).

**TABLE 3:** Perceptual responses during sets 1, 2, and 3 of repeated‐sprint exercise in the four conditions.

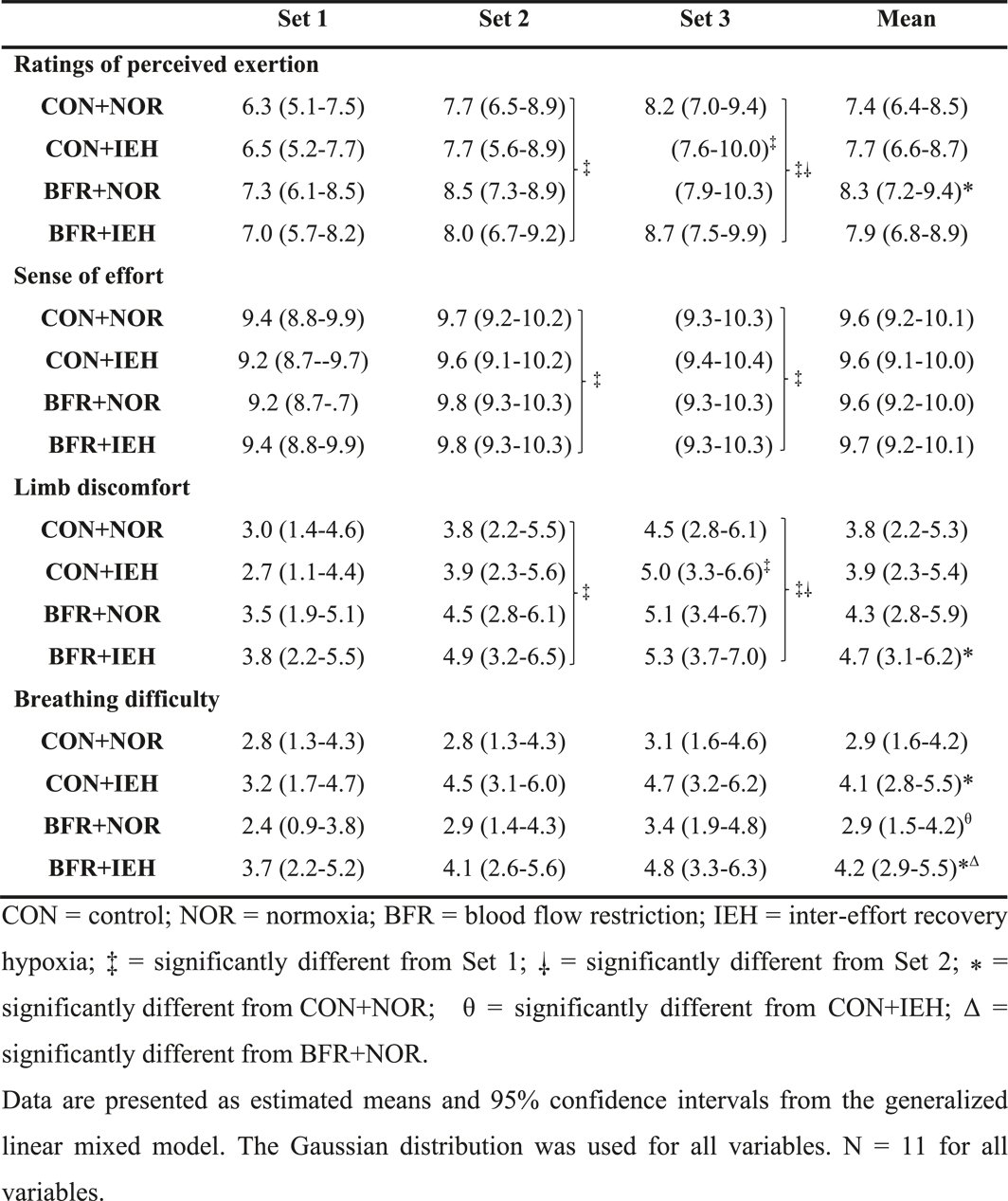

Sense of effort showed a significant main effect of set. Post hoc analyses indicated a higher sense of effort in Sets 2 and 3 compared to Set 1 (*p* < 0.001; *d* > 0.743; Table 3).

Limb discomfort showed a significant main effect of set and condition. Post hoc analyses indicated that limb discomfort increased as the sets progressed (*p* < 0.047; *d* > 0.739; Table 3), and it was higher in BFR + IEH than in CON + NOR (*p* = 0.037; *d* = 0.840; Table 3).

Breathing difficulty also exhibited a significant main effect on the condition. The IEH presented higher breathing difficulty compared to the NOR conditions (*p* < 0.007; *d* > 0.998; Table 3).

## 4 Discussion

### 4.1 Summary of Main Findings

This study compared the acute effects of combining BFR during sprints with IEH on RSE performance and psycho‐physiological responses. The main findings were: (i) power output decreased across sets with an impairment in peak power caused by BFR; (ii) VO_2_ and ventilation during the recovery between sets were higher, while SpO_2_ was lower in IEH compared to NOR conditions; (iii) HR and [La] were not influenced by either BFR or IEH; (iv) BFR and IEH did not affect the perceptual measurements. Altogether, these data suggest that combining BFR during sprints with IEH is a viable strategy to maintain power output while imposing additional physiological stress. Our hypotheses were largely supported, except that BFR did not accentuate local deoxygenation or exercise‐related sensations, as expected.

### 4.2 Mechanical Output

Power output declined across sets, likely due to the accumulation of central and peripheral fatigue (Hureau et al. 2016). Local hypoxia caused by BFR may impair oxygen delivery and phosphocreatine resynthesis (Harris et al. 1976), and continuous BFR application has been shown to reduce RSE performance (Mckee et al. 2023; Peyrard et al. 2019; Solsona et al. 2024; Wizenberg et al. 2023). However, consistent in part with Mckee et al. (2023), our results show that BFR applied exclusively during the sprints does not compromise MPO and work but affects PPO in Set 3. Likewise, systemic hypoxia impairs RSE performance only when applied continuously (Li et al. 2024; Peyrard et al. 2019; Solsona et al. 2024; Takei et al. 2024). However, IEH has been shown not to affect performance in both high‐intensity interval training sessions (Dellavechia de Carvalho, Ferronato, et al. 2023; Dellavechia de Carvalho, Ferronato, et al. 2023; Foresti et al. 2023) and RSE (Li et al. 2024), corroborating our results. Additionally, Botta de Arruda et al. (2025) showed greater power output in a sprint interval exercise session, in the IEH condition compared to normoxia. Therefore, combining BFR during sprints and IEH appears to preserve performance and does not have any cumulative effect, making it a promising strategy for repeated‐sprint training.

### 4.3 Physiological Responses

Regarding cardiorespiratory responses, BFR had no significant effect. In contrast, VO_2_ and ventilation were higher, and SpO_2_ lower, in IEH compared to NOR conditions, whereas [La] and HR did not differ between conditions. The impact of BFR on cardiorespiratory responses remains debated. Solsona et al. (2024) reported reductions in VO_2_ and ventilation with continuous BFR, whereas others found no such effects (Mckee et al. 2023; Willis et al. 2018; Willis et al. 2019). Our findings align with those of Mckee et al. (2023), who showed that BFR applied solely during sprints does not alter ventilatory responses during RSE.

Continuous systemic hypoxia has consistently been associated with reduced ventilatory responses during RSE (Solsona et al. 2024; Yamaguchi et al. 2021). However, our study is the first to document ventilatory responses under IEH. Interestingly, both VO_2_ and ventilation during recovery were higher in IEH than in NOR, likely due to preserved mechanical performance and sustained energy demand. The elevated VO_2_ may also reflect hypoxia‐induced compensatory vasodilation, possibly mediated by α‐adrenergic receptor blockade (Casey and Joyner 2012; Umbrello et al. 2013).

As expected, SpO_2_ was lower during IEH recovery periods, aligning with previous studies (Dellavechia de Carvalho, Ferronato, et al. 2023; Dellavechia de Carvalho, Ferronato, et al. 2023; Foresti et al. 2023). No significant differences in HR were noted between the conditions. Nonetheless, the effect of IEH on HR remains unclear, because our previous studies showed higher HR under the IEH condition (Botta de Arruda et al. 2025; Foresti et al. 2023), and others did not (Dellavechia de Carvalho, Ferronato, et al. 2023; Dellavechia de Carvalho, Ferronato, et al. 2023). High inter‐individual variability and the small effect size of IEH on HR may explain the lack of difference. Likewise, neither BFR during sprints (Mckee et al. 2023) nor IEH (Botta de Arruda et al. 2025; Dellavechia de Carvalho, Ferronato, et al. 2023; Dellavechia de Carvalho, Ferronato, et al. 2023; Foresti et al. 2023) altered blood lactate accumulation, suggesting that [La] is more strongly tied to exercise intensity than oxygen availability. Overall, our results show that IEH alone induces additional physiological stress during RSE, independent of BFR (during sprints) application.

### 4.4 Muscle Oxygenation

This study demonstrates that applying BFR exclusively during sprint efforts does not significantly alter muscle oxygenation profiles or impair sprint power output. In contrast, previous studies employing intermittent BFR during interset (3 sets of 3 x 6‐s sprints with 24 s recovery) (Ienaga et al. 2022) or intraset recovery (5 × 10‐s sprints with 40 s recovery) (Kojima et al. 2021) periods have reported marked muscle deoxygenation, yet similarly observed no detriment in mechanical power. Continuous application of BFR during RSE also elicits marked muscle deoxygenation, but coincides with reduced sprint performance, indicating a trade‐off in absolute exercise intensity (Mckee, Girard, Peiffer, Dempsey, et al. 2024). Collectively, these findings indicate that intermittent BFR, whether applied during efforts or recovery, may preserve absolute training intensity, thereby maximizing interval training benefits (Papoti et al. 2023). However, the current findings indicate that BFR during sprint efforts alone may be insufficient to elevate metabolic stress, as muscle oxygenation remained unperturbed. The brief ischemic stimulus (i.e., 5 s) is likely mitigated by relatively long unrestricted recovery periods (i.e., 25 s), maintaining the balance between O_2_ demand and supply. Another possible explanation relates to the body position used during AOP assessment. Previous studies have shown that AOP determined in the supine position may underestimate the pressure required during seated or standing exercise conditions (Hughes et al. 2018; Karanasios et al. 2022; Sieljacks et al. 2018). Consequently, the effective restrictive stimulus during exercise may have been lower than intended. Future research should determine AOP in the exercise‐specific body position and examine whether longer print durations or shorter recovery intervals increase the physiological load imposed by this BFR modality.

The present study also reports that TSI values remained lower during inter‐set recovery when IEH was applied. This is likely driven by the reduced inspired O_2_ partial pressure, limiting alveolar and skeletal muscle O_2_ availability (Gore et al. 2007). Despite this attenuated recovery, muscle oxygenation during subsequent sprint sets remained comparable across all conditions. This may be due to the rapid wash‐out of hypoxic air upon re‐exposure to normoxia, allowing oxygen delivery and utilization to normalize before the next set.

### 4.5 Exercise‐Related Sensations

Across all sets, RPE, sense of effort, and limb discomfort progressively increased, regardless of condition, consistent with previous studies employing comparable RSE protocols (Li et al. 2024; Mckee et al. 2023). The sense of effort, despite the increase after Set1, remained stable, indicating that participants maintained near‐maximal effort across all sprints and could distinguish between task‐specific effort and overall exertion (i.e., RPE). This is consistent with a previous study (Girard, Billaut, et al. 2017) showing increased overall exertion during RSE (ten 4‐s maximal sprints with 30 seconds rest), while the sense of effort stayed near maximal. Li et al. (2024) reported that IEH did not affect RPE and leg discomfort but increased difficulty breathing under continuous exposure, corroborating our findings. Furthermore, Mckee et al. (2023) found that continuous BFR and BFR applied during recovery increased RPE and limb discomfort, whereas BFR applied exclusively during efforts did not; however, our results showed higher RPE with the BFR only during efforts, so this effect remains unclear. Notably, combining both (i.e., BFR + IEH condition) had no additive effect on the sense of effort, breathing difficulty, and RPE, this last being used in the subjective assessment of internal training load (Foster et al. 2001), but increased limb discomfort.

#### 4.5.1 Limitations

This study provides important information about the combination of local and systemic hypoxia, but it is not without limitations. Only one BFR pressure (45% AOP) was investigated, based on previous pilot work showing that this was the highest pressure tolerable during intermittent BFR exercise (Mckee, Girard, Peiffer, Dempsey, et al. (2024). Furthermore, the pressure applied to both legs was determined from AOP assessment of the right leg only, despite the potential for inter‐limb variability in AOP. The target pressure used during the experimental sessions was based on measurements obtained during the familiarization session; therefore, day‐to‐day physiological variability in AOP and cuff placement may have influenced the restrictive stimulus. Furthermore, AOP was assessed with participants in the supine position, which may have underestimated the pressure required during the experimental exercise conditions. Similarly, the systemic hypoxia dose is critical (Millet et al. 2016), and while different FiO_2_ levels may be more effective, there is currently no evidence identifying what hypoxia dose is best for implementing the IEH strategy. The participants were healthy, active young adults, limiting the generalizability of these findings to lower or better‐trained cohorts. Sex differences in metabolic and cardiovascular responses to exercise and hypoxia suggest caution when applying these results to women (Raberin et al. 2024). There are practical limitations in the blinding process for both BFR and systemic hypoxia as these interventions may be perceptible to participants and could therefore introduce expectancy effects, although some participants reported difficulty distinguishing between conditions. Future studies should also consider the use of forehead, ear, or reflectance sensors to improve the accuracy of SpO_2_ measurements during maximal‐intensity exercise. NIRS‐derived measures were obtained unilaterally from the right vastus lateralis and therefore reflect local, site‐specific changes in muscle oxygenation. Caution is warranted when extrapolating these findings to whole‐limb or bilateral muscle responses. In addition, NIRS signals may be influenced by probe placement and adipose tissue thickness although probe positioning was standardized across conditions. Finally, given the growing use of repeated‐sprint training in hypoxia (systemic and/or local hypoxia), further investigation into the underlying fatigue mechanisms is needed. Utilizing tools such as EMG to assess neural drive and percutaneous nerve stimulation to pinpoint fatigue origin could help refine best practices in this area.

#### 4.5.2 Practical Implications

Applying BFR during efforts and IEH does not compromise performance in an RSE acute session, yet both impose higher physiological stress during the session. This provides practitioners and coaches with a useful strategy to enhance physiological stimulus through local or systemic hypoxia without altering mechanical output. Using one or both strategies may achieve similar cardiometabolic outcomes at lower intensities, helping to manage overload and shorten recovery time. Alternatively, they can enhance benefits at the same intensity, which could lead to better performance outcomes over time.

## 5 Conclusion

Despite heightened physiological responses, including higher VO_2_ and ventilation together with lower SpO_2_ and mean TSI in IEH, no detectable impairment in mechanical output was observed with this specific BFR dosage (45% AOP during 5‐s sprint efforts) combined with systemic hypoxia during recovery in healthy, physically active individuals. These findings may not necessarily generalize to other BFR pressures, sprint durations, or recovery structures.

## Funding

This study was possible thanks to the Federal Agency for Support and Evaluation of Graduate Education (Coordenação de Aperfeiçoamento de Pessoal de Nível Superior, CAPES) through the programme CAPES‐PrInt (88887.835398/2023‐00). Authors CDC, GMP, and MP are supported by São Paulo Research Foundation (Fundação de Amparo à Pesquisa do Estado de São Paulo, FAPESP) through the grants Thematic project (2023/02728‐3) and Technical training (2026/02573‐8). Author BRS is supported by an Investigator Grant from the National Health and Medical Research Council (GNT1196462).

## Ethics Statement

All participants completed the Exercise & Sports Science Australia (ESSA) pre‐exercise screening and provided written consent. The study was approved by The University of Western Australia Human Research Ethics Committee (2023/ET000791). All sessions were conducted at the UWA Exercise & Performance Center, located at sea level.

## Consent

Upon arrival, participants provided informed consent and completed Part 1 of the ESSA pre‐exercise screening. All cycling tests were conducted on a custom‐made front‐access cycle ergometer (School of Sport Science, Exercise and Health; University of Western Australia), calibrated using the NATA Ergometer Calibration Rig (Biomed Electronic Services, Australia). Seat height, handlebar position, and gear range were individually adjusted during familiarization and replicated in all trials. Mean power output (MPO), peak power output (PPO), and total work were recorded for each sprint at 1 Hz using customized software (Cyclemax, The University of Western Australia, Australia).

## Conflicts of Interest

The authors declare no conflicts of interest.

## Data Availability

The data that support the findings of this study are available from the corresponding author upon reasonable request.
